# Adoption of a Newly Introduced Dermal Matrix: Preliminary Experience and Future Directions

**DOI:** 10.1155/2020/3261318

**Published:** 2020-10-16

**Authors:** Andrea Vittorio Emanuele Lisa, Leonardo Galtelli, Valeriano Vinci, Alessandra Veronesi, Luca Cozzaglio, Ferdinando Carlo Maria Cananzi, Federico Sicoli, Marco Klinger

**Affiliations:** ^1^Plastic Surgery Unit, Department of Medical Biotechnology and Translational Medicine BIOMETRA, Humanitas Clinical and Research Hospital, Reconstructive and Aesthetic Plastic Surgery School, University of Milan, Via Manzoni 56, Rozzano, Milan 20090, Italy; ^2^Department of Biomedical Sciences, Humanitas University, 20090 Pieve Emanuele, Italy; ^3^Sarcoma, Melanoma and Rare Tumors Surgery Unit, Humanitas Clinical and Research Center, Via Manzoni 56, 20089 Rozzano, Italy

## Abstract

**Introduction:**

Acellular dermal matrix (ADM) products are adopted in the management of injuries to soft tissues. ADMs have been increasingly employed for their clinical advantages, and they are acquiring relevance in the future of plastic surgery. The aim of our study is to evaluate the application of ADMs in our patients who could not undergo fast reconstruction.

**Materials and Methods:**

We performed a retrospective study on 12 patients who underwent ADM placement for scalp and limb surgical reconstructions at the Humanitas Research Hospital, Rozzano (Milano), Italy. Wounds resulted from 9 tumor resections and 3 chronic ulcers. The ADM substrate used to treat these lesions was PELNAC™ (Gunze, Japan), a double-layered matrix composed of atelocollagen porcine tendon and silicon reinforcement. All patients underwent a second surgical operation to complete the treatment with a full-thickness skin graft to cover the lesion.

**Results:**

In this study, 12 patients were treated with PELNAC™: 11 out of 12 patients showed a good attachment over a median time of 21.3 days (range 14-27). After almost 23 days, all patients were ready to undergo a full-thickness skin grafting.

**Conclusion:**

This study assesses the benefits of PELNAC™ and proposes this method as an alternative to traditional approaches, especially in situations where the latter techniques cannot be applied.

## 1. Introduction

Acellular dermal matrix products are used in the management of soft tissue injures, resulting from either acute or chronic conditions such as mechanical traumas, burns, ulcers, or tumors.

Acellular dermal matrices (ADMs) were initially described to be used in the resurfacing of burn injuries, abdominal wall repair, tympanic membrane replacement, dural repairs, and gingival grafting. In the field of breast surgery, ADMs were first introduced in revisional aesthetic surgery, including correction of implant rippling, symmastia, and soft tissue deficits [1, 2].

In recent years, ADMs have been increasingly employed for their clinical advantages. It is reported that the decellularised matrices of ADM integrate with the host tissue wounds, resulting in the regeneration and the revascularization of the structure, leading to normal skin regeneration [3, 4].

The development of new materials allows for continuous renewal of many procedures. ADM use is thus acquiring an important position in the future of plastic surgery.

Integra® Dermal Regeneration Template (Life Sciences, Plainsboro, NJ, USA) is a bilayer skin substitute. The “dermal” (lower) layer is a bovine collagen base with glycosaminoglycan chondroitin-6-sulfate while the upper layer is a silicone sheet that acts as a temporary epidermis. As the wound heals, the dermal layer is replaced by the patient's own cells. A thin split-thickness skin graft is successively applied on the neodermis. Integra is indicated for the management of complex wounds such as partial- or full-thickness burns and multiple types of ulcers [5, 6]. GraftJacket® (Wright Medical Technology, Arlington, TN, USA) is derived from donated human cadaveric skin. After a first screening for HIV, syphilis, human T-lymphotropic virus (HTLV; types 1 and 2), and hepatitis B and C, the antigens from donor cells are removed to reduce histochemical reactions. Scientific literature reports its effective use in treating diabetic foot ulcer [7]. Matriderm® (Skin and Health Care AG, Billerbeck, Germany) is a 3-dimensional matrix composed of intact collagen fibrils types I, III, and V and alpha-elastin of bovine origin. Matriderm can be employed in a one-stage procedure with a split-thickness skin graft, and its use may minimize the loss of pliability and elasticity of the skin [8].

Since researches are increasingly offering the best materials in terms of quality and costs, we want to illustrate our preliminary experience in wound healing processes with the use of PELNAC™ (Gunze, Japan), a new type of acellular dermal matrix, recently introduced in our clinical practice for the management of chronic wounds in different locations, caused by inadequate vasculature, diabetes, infections, traumas, or tumors.

The aim is to evaluate PELNAC™ (Gunze, Japan) effectiveness in patients who could not be reconstructed immediately, but needed a “bridge treatment” before being grafted.

## 2. Materials and Methods

We performed a retrospective study on patients treated surgically at the Humanitas Research Hospital, Rozzano (Milano), Italy, from October 2018 to October 2019.

A first screening included a comprehensive physical examination and documented medical history.

The complete inclusion and exclusion criteria applied to assess eligibility of patients to therapy are listed in Table 1, while patients' characteristics are reported in Table 2.

Clinical evaluation was based on two main parameters: percentage of engrafting and development of complications (e.g., infection) during the wound healing process.

## 3. Procedure

All patients agreed to participate in the study by written informed consent. The ADM substrate used was PELNAC™ (Gunze, Japan), which is a temporal dermal matrix used for wound treatment. The substrate is double layered and composed of atelocollagen porcine tendon and silicon reinforcement. All surgical procedures were performed in the operating room.

Debridement of damaged host tissue was performed along with tissue hemostasis and washing procedures. The substrate was trimmed in order to reach a better adherence according to wound margins.

**Figure 1:**
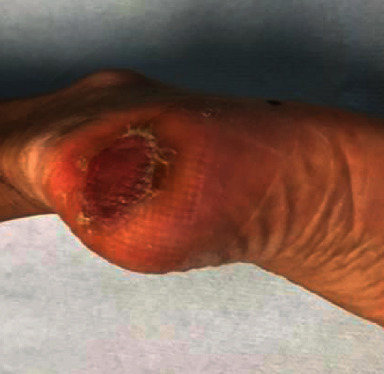
Postoperative picture (29 days after first operation and 7 days after skin grafting) with total resolution of lesion.

PELNAC™ (Gunze, Japan) was then immersed in saline solution before being applied as coverage. Afterwards, the ADM was placed on the damaged area and it was secured to the host healthy skin by means of few sutures. PELNAC™ (Gunze, Japan) presents a fenestrated matrix for some sheets without the need to perform any cut to facilitate spill of fluids collected between the host tissue and the applied collagen layer. Then, an external dressing was applied and replaced every week during ambulatory visits, checking for the presence of infection. Once the patient was ready, after a mean of three weeks, the silicon film was peeled off and a second surgery was performed to complete the treatment with a full-thickness skin graft (Figure 1).

## 4. Results

In this study, 12 patients were treated with PELNAC™ (Gunze, Japan) for scalp and limb surgical reconstruction, covering wounds ranging from 4.5 to 10 cm^2^ (median of 7.5 cm^2^). Eleven patients out of 12 had a successful attachment of the matrix (percentage of attachment 91.7%), over a time frame ranging from 14 to 27 days (median 21.3 days).

**Figure 2:**
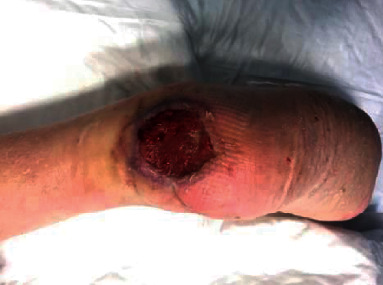
Postoperative picture (22 days postop); matrix is engrafted with no signs of infections.

We observed a partial failure in 1 patient. No infections were observed (Figure 2).

**Figure 3:**
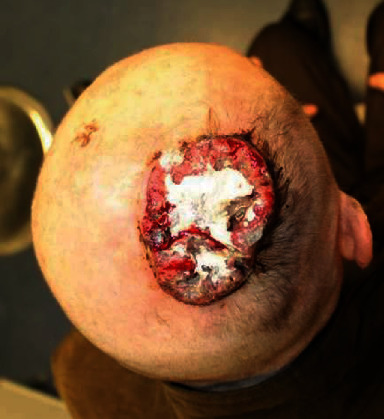
Preoperative picture of a skin cancer of the scalp region in a 73-year-old patient.

**Figure 4:**
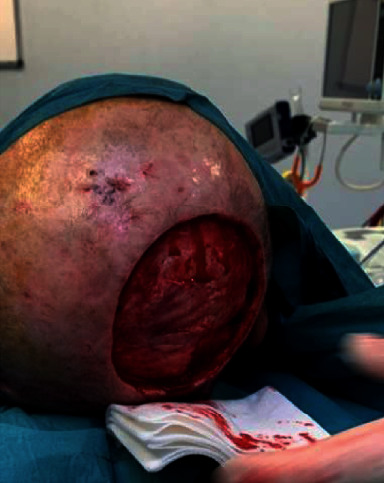
Intraoperative picture after complete surgical and fascial removal. Before PELNAC placement (histology—squamous cell carcinoma).

**Figure 5:**
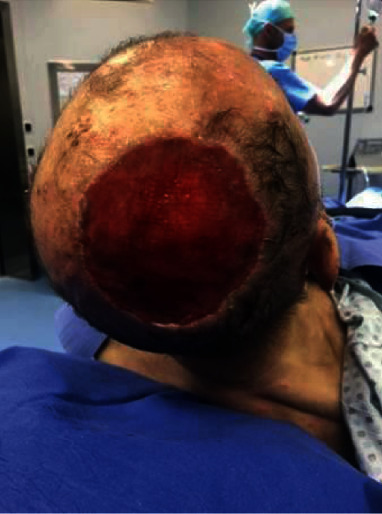
Acellular dermal matrix grafted 21 days after first time operation before being skin grafted.

**Figure 6:**
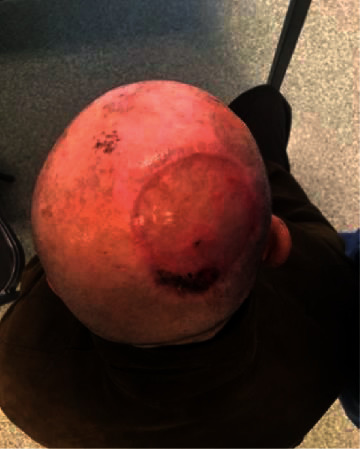
Postoperative picture 28 days after surgery. A small dehiscence is observed in the posterior side which subsequently resolved after 7 days.

After almost 23 days, all patients were ready to undergo a full-thickness skin grafting procedure to complete the treatment (some cases are reported in Figures 3–6).

## 5. Discussion

The use of skin substitutes has increased over the past decades for the treatment of a wide range of acute and chronic limb injuries. Biologic, synthetic, or biosynthetic tissues have been developed, and it is crucial to understand their properties and composition for an optimal use in promoting coverage and regeneration of tissues from open wounds [1].

The Davison-Kotler classification groups all tissue substitutes based on different elements: material composition, time of permanence, intended layer of replacement, and presence or lack of cellular component [9].

A specific group of composites includes ADM, a substrate characterized by the absence of cellular components. Absence of cells leads to major clinical implications regarding tissue application, storage, cost, and availability of the product. ADM results in being less reactive to host immune cell reaction compared to cellularised products. Furthermore, cellularised products are more difficult to produce and more expensive with respect to ADMs.

Such advantages have led to the development of different ADM products which differ in layering, material, and permanence. As a consequence, the latter are adopted under specific conditions.

The purpose of this study is to report the use of PELNAC™ (Gunze, Japan) in patients with scalp and limb injuries.

The product is made of atelocollagen-derived porcine tendon and a reinforced silicone layer. It promotes skin regeneration, and it is gradually replaced by host tissues as the atelocollagen structure is revascularized with fibroblasts and capillary infiltrates. In our experience, we did not detect any infection. Indeed, after grafting, we observed minimal skin contracture and normal pigmentation.

The main advantage of this ADM is the presence of several types of materials with their own characteristics: a fortified one made of a two-layered material as described above, but there are also others with stronger (4.5x) silicone film layer that allows for suture or stable fixation, and a fenestrated matrix type which has the characteristic of being meshed allowing for better wound conformity and drainage.

Literature confirms that the use of PELNAC™ (Gunze, Japan) improves healing processes in chronic injuries and increases the patient's overall satisfaction [10].

It is not always easy to identify objective elements that support outcomes of the usage of these materials; thus, we collected preoperative and postoperative pictures to show results of our own cases.

Under the sustainability aspect, we observed a reduction in costs mainly due to the fact that this material is less expensive than other skin substitutes present on the market.

**Figure 7:**
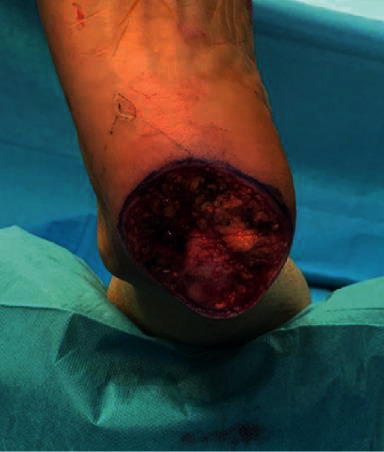
Operative picture: removal of a melanoma of the right calcaneal region (acral melanoma in vertical growth, Clark IV, Breslow 2.5mm).

In contrast to other materials, PELNAC™ (Gunze, Japan), being made of a layer of fenestrated atelocollagen, is a perfect solution for reconstructive surgery in lower limbs, even more in areas where skin folds complicate the surgery (Figure 7). In such areas, small fragments of the atelocollagen can be used to make the surface as plane as possible in order to better engraft the matrix.

In particular, our team treated a patient with an interdigital lesion after melanoma resection using this method; the aforementioned area is particularly difficult to treat, although it healed properly after being grafted.

The only failure we observed was a patient who underwent a surgical intervention to excise a scalp squamous cell carcinoma followed by a full-thickness graft reconstruction after 21 days. After 7 days, a dehiscence appeared; however, it was successfully solved thanks to follow-up medications (Figure 6).

This complication is commonly caused by a local traumatism, especially in patients without any other systemic or local risk factor of failure.

The main limits of our study are the limited number of cases and the fact that we built a retrospective study.

## 6. Conclusion

This study describes our experience using PELNAC™ (Gunze, Japan) for scalp and limb injuries.

We had the chance to assess benefits of PELNAC™ (Gunze, Japan), and we may propose this method as an alternative to traditional approaches, especially in situations where the latter techniques cannot be applied.

PELNAC™ (Gunze, Japan) used as a temporary dermal matrix was beneficial to patients not only for the quality of reconstruction itself but also in every single collateral aspect of the intervention, including postoperative pain allowing for a quick recovery from the procedure and absence of complications.

The present study has highlighted interesting results on the usage of this dermal matrix. Therefore, our research will be extended in the future to a larger group of patients.

## Figures and Tables

**Table 1:** Inclusion and exclusion criteria.

Inclusion criteria	Exclusion criteria
Wound presence > 4 weeks (chronic wound)	Wound treated with a biomedical or topical growth factor within 30 days
Low vascularization within the prior 60 days	Index wound > 25 cm^2^
Tumor resection	Serum creatinine > 3.0 mg/dL
Patient > 18 years old	Known history of poor compliance to medical or surgical treatments
Wound larger than 1 cm^2^, noninfected	Ongoing radiotherapy or chemotherapy
Pathology which compromises blood supply or healing (diabetes, smokers, and radiotherapy)	Autoimmune diseases
	Pregnancy or breastfeeding

**Table 2:** Population's characteristics.

Population's characteristics	
Sex	5 women; 7 men
Age (median)	70 years (range 35-82)
Primary pathology	8 skin cancers; 4 chronic ulcers
Wound site	4 scalp region; 8 lower limbs
Wound extension (median)	7.5 cm^2^ [4–10]

## Data Availability

Data can be provided by authors on demand at any time.
